# Non-Seasonal Variation of Airborne *Aspergillus* Spore Concentration in a Hospital Building

**DOI:** 10.3390/ijerph121113730

**Published:** 2015-10-28

**Authors:** Michael Oberle, Markus Reichmuth, Reto Laffer, Cornelia Ottiger, Hans Fankhauser, Thomas Bregenzer

**Affiliations:** 1 Kantonsspital Aarau AG, Institute for Laboratory Medicine, Tellstrasse 1, 5001 Aarau, Switzerland; E-Mails: cornelia.ottiger@ksa.ch (C.O.); hans.fankhauser@ksa.ch (H.F.); 2 Analytica Medizinische Laboratorien AG, Falkenstrasse 14, 8024 Zürich, Switzerland; E-Mail: m.reichmuth@analytica.ch; 3 Spital Langenthal, Medizinische Klinik, St. Urbanstrasse 67, 4901 Langenthal, Switzerland; E-Mail: r.laffer@sro.ch; 4 Spital Lachen, Klinik für Innere Medizin, Oberdorfstrasse 41, 8853 Lachen, Switzerland; E-Mail: thomas.bregenzer@spital-lachen.ch

**Keywords:** *Aspergillus*, airborne fungal spores, seasonal variation, spore contamination, hospital air, monitoring

## Abstract

Nosocomial fungal infections are gaining increased attention from infectiologists. An adequate investigation into the levels of airborne *Aspergillus* and other fungal spores in hospital settings, under normal conditions, is largely unknown. We monitored airborne spore contamination in a Swiss hospital building in order to establish a seasonally-dependent base-line level. Air was sampled using an impaction technique, twice weekly, at six different locations over one year. Specimens were seeded in duplicate on Sabouraud agar plates. Grown colonies were identified to genus levels. The airborne *Aspergillus* spore concentration was constantly low throughout the whole year, at a median level of 2 spores/m^3^ (inter-quartile range = IQR 1–4), and displayed no seasonal dependency. The median concentration of other fungal spores was higher and showed a distinct seasonal variability with the ambient temperature change during the different seasons: 82 spores/m^3^ (IQR 26–126) in summer and 9 spores/m^3^ (IQR 6–15) in winter. The spore concentration varied considerably between the six sampling sites in the building (10 to 26 spores/m^3^). This variability may explain the variability of study results in the literature.

## 1. Introduction

Data on the frequency and distribution of *Aspergillus* and other fungal spores in hospital buildings, under normal conditions, are insufficient to lead to meaningful conclusions [1,2]. Samplings of airborne spores are often done under specific conditions, such as an increased rate of invasive aspergillosis (IA), to identify the sources or reasons for the contamination. To better estimate the situation under investigation, an in-house spore concentration base-line is necessary for comparison with buildings with mould related problems. 

Inhaled spores of *Aspergillus*, otherwise considered harmless to an immune competent individual, can cause life threatening invasive fungal infections in immune-suppressed patients, such as haematooncological patients or in patients after solid organ transplantation. Management of fungal infections is challenging, treatments are expensive, and morbidity and mortality rates are high [3,4]. The accommodation of highly susceptible patients in HEPA air-filtered units is, therefore, an internationally accepted preventive measure [5,6].

The levels of inhalable fungal spores are increased in outdoor air compared to indoor air, and during summer compared to winter (season dependent) [7,8,9]. Factors, such as construction or demolition activities, *i.e.*, increased dust production [10], potted plants [11], humidifier, stagnant water, humidity, or condition of the building influence the spore concentration. These diverse factors explain why sources of fungal spore contamination are often difficult to find and remain unidentified in more than 20% of studies [12].

Reliable data on the levels and seasonal distribution of airborne *Aspergillus* and other fungal spores in hospital settings under normal conditions are pertinent. In this study, we monitored, for one year, the airborne fungal spore contamination in one building of the Cantonal Hospital of Aarau, Switzerland.

## 2. Materials and Methods

The study-site was in a building of the Cantonal Hospital of Aarau (altitude: 380 meters above sea level), in central Switzerland, constructed in 1996. An outpatient clinic is located on the ground level of the building, and on floors 1 to 5 are wards with a total of 142 beds. A 4-bed unit for protective isolation of patients with long-lasting neutropenia, due to induction and consolidation chemotherapy for leukemia, is located on the 4th floor. The rooms of this unit are kept under positive HEPA-filtered air-pressure through an air-conditioner. The hospital (the remaining rooms, corridors and stair case) has no ventilation system.

One month prior to the sampling, all soil-plants were removed from the building. The monitoring was done twice weekly at 6 sampling sites in the building (started in September 2008 until August 2009). The sampling sites were in the staircase and in the recreation area of the following levels: In the basement close to the main entrance, where the influence of the incoming outdoor air on the hospital air quality was of interest. On the 4th floor, where immune-suppressed patients are treated and the HEPA equipped unit is located. On the 5th floor, as a control to the 4th floor, far away from the main entrance and where the influence of outdoor air is marginal.

Air sampling was done 1.05 meters above ground with a MAS100 (Microbiological air sampler, MBV AG, Stäfa, Switzerland) using the impaction principle on a 90 mm Sabouraud dextrose agar plate through a sieve with 400 holes. Air intake velocity was 100 L air per minute. Each plate was seeded for five min, resulting in an air-volume of 500 L. Impacted spores grew to visible colonies on the agar plate. Errors due to more than one spore simultaneously passing through a hole and produce one colony only were corrected using a MBV AG calculation table (according to the manufacturers instruction). The corrected number colonies multiplied by two resulted in the concentration of spores per cubic meter (spores/m^3^) [13]. To take advantage of the air agitation due to health care workers, patients, and visitors, the air samplings were conducted in the afternoon, during normal hospital activities. Temperature and humidity in the building were recorded at each sampling date. Exceptional events (e.g., open windows, *etc.*) were also recorded. 

Two Sabouraud dextrose agar (SAB) plates were seeded at each site. One of these was incubated for 4 days at 28 °C to allow growth of all cultivatable fungal spores, representing the local biodiversity. The other was incubated at 36 °C for 2 days to detect the thermotolerant *Aspergillus* spp. These aspergilli play an important role for IA since thermotolerance in *A. fumigatus* was shown to be linked to fungal pathogenesis [14]. Colonies of fungi were identified macro- and microscopically at the genus level. Lactophenol blue stained Scotch-preparations were done for the microscopic analyses.

The climate data were received from the weather station Buchs/Aarau (about 1 km east of the hospital), which belongs to the Federal Office for Meteorology and Climatology of Switzerland [15]. Daily means of air temperature, relative humidity (measured 2 meter above ground), and precipitation were used to calculate the monthly geometric mean for this study. Two seasons, winter (December to May) and summer (June to November), were specified. 

Statistical analysis was done with SPSS (version 20.0). Comparison of the non-parametrically distributed spore concentrations in winter and summer was done with Mann-Whitney-U two-tailed test. The Wilcoxon-test was used to analyze spore distribution in the building. *p* < 0.05 is considered as significant.

## 3. Results

The Hospital indoor air was constantly contaminated with a concentration of two spores of *Aspergillus* spp*.* per m^3^ (IQR 1–4) throughout the year. This corresponds to a frequency of 6.7% of all fungal spores (Table 1). *Aspergillus* spores showed no seasonal variation between winter and summer. Seventy-seven percent of all *Aspergillus* spp. grew at 36 ± 1 °C (thermotolerant isolates) of which more than 95% were identified as *A. fumigatus*. The remaining genera identified were: *Alternaria*, *Cladosporium*, *Mucor/Rhizopus*, and *Penicillium*. The genera, *Chrysonilia*, *Fusarium*, *Paecilomyces*, *Scopulariopsis*, *Trichoderma*, and unidentified molds are summarized as “others” (Table 1). *Alternaria* (frequency of 2.9%) and especially *Cladosporium* (80.1%) showed a significant seasonal variability whereas the remaining groups did not show any significant seasonal dependency (Table 1).

**Table 1:** Number and frequency of fungal genera (left) and their seasonal medians and inter-quartile range (IQR). Inter-seasonal concentration was compared using Mann-Whitney-U test (*p* < 0.05 is considered as significant). Records collected on 5 January 2010, when Christmas trees were removed, are excluded from the analysis. Thermotolerant *Aspergillus* is shown separately in the row at the bottom.

Fungal Genus	Total Colonies		Summer		Winter		*p*
	Number	Frequency	Median (spores/m^3^)	IQR	Median (spores/m^3^)	IQR	
*Aspergillus* total	1144	6.7%	2.3	1–4.6	1.8	0.8–4.2	0.26
*Alternaria*	494	2.9%	0.3	0.3–1.1	0.3	0.3–0.3	<0.001
*Cladosporium*	13,618	80.1%	23.5	7–134	1	0–2	<0.001
*Mucor/Rhizopus*	78	0.5%	0	0–0.3	0	0	0.053
*Penicillium*	1149	6.8%	2.4	1.6–4.1	3.2	1.3–5.6	0.77
Others	516	3%	0.2	0–0.3	0.2	0–0.5	0.71
Total	16,999	100%	82	26.3–126	9	6–15	<0.001
Thermotolerant *Aspergillus* spp.	881	77% (of all *Aspergillus*)	2.2	1–3.4	1.8	0.8–4.3	0.54

**Figure 1:**
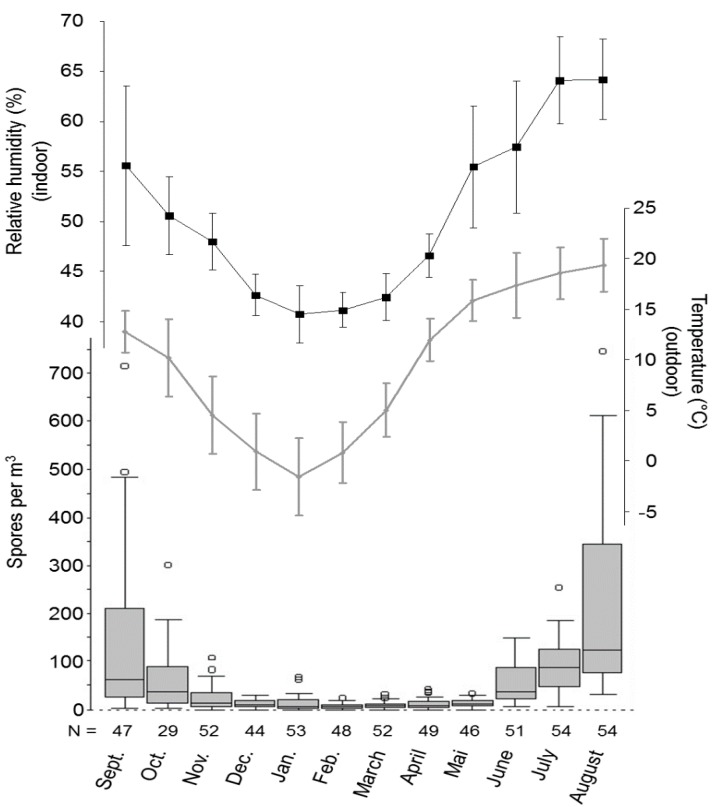
Dynamics of the monthly median of fungal spores concentrations, the outdoor mean temperatures and the indoor mean relative humidity (*N* = number of Sabouraud Petri dishes analyzed, sampling twice a week).

Figure 1 summarizes the monthly indoor median spore concentration throughout the year which parallels the dynamic of the mean temperature measured outdoor, as well as the relative humidity measured in the hospital building. The mean temperature in winter was 5.5 °C (SD 6.4) and in summer 13.8 °C (SD 5.3). The mean indoor relative humidity was 44.8% (SD 5.1) in winter and 65.8% (SD 6.1) in summer. The temperature inside the building was constantly 24 °C (SD 1.2) and showed no seasonal variability.

**Figure 2:**
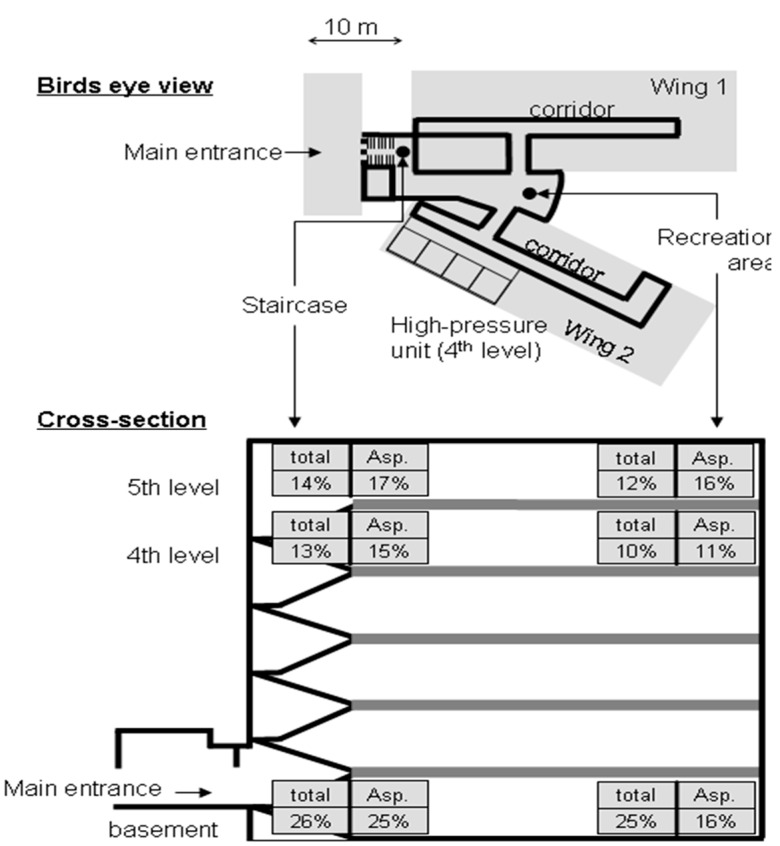
Overview of the study site: Birds eye view (**upper** **panel**): The black points show the sampling locations in the stair case and the recreation room and on the 4th level the HEPA air-filtered high pressure unit. Cross-section of the building (**lower** **panel**): The six sampling sites are indicated with grey boxes. The detection rates, in percentages, of all cultivable fungi (total) or *Aspergillus* spores (Asp.) are displayed.

The highest recovery rate of *Aspergillus* spores was in the staircase close to the main entrance of the building (25%) and the lowest rate in the recreation area on the 4th level (11%) next to the HEPA filtered units (Figure 2). The *Aspergillus* spore concentrations at these two sites were significantly diverse (*p* < 0.001, Wilcoxon-test) compared to the median in-house concentration. Again, the recovery rate of the remaining spores was highest in the basement and low in the upper levels. The significant lowest recovery rate of the remaining fungal spores was, similar to *Aspergillus* spores, in the recreation area on the 4th level (10%) next to the HEPA filtered rooms (*p* < 0.001, Wilcoxon-test).

**Figure 3:**
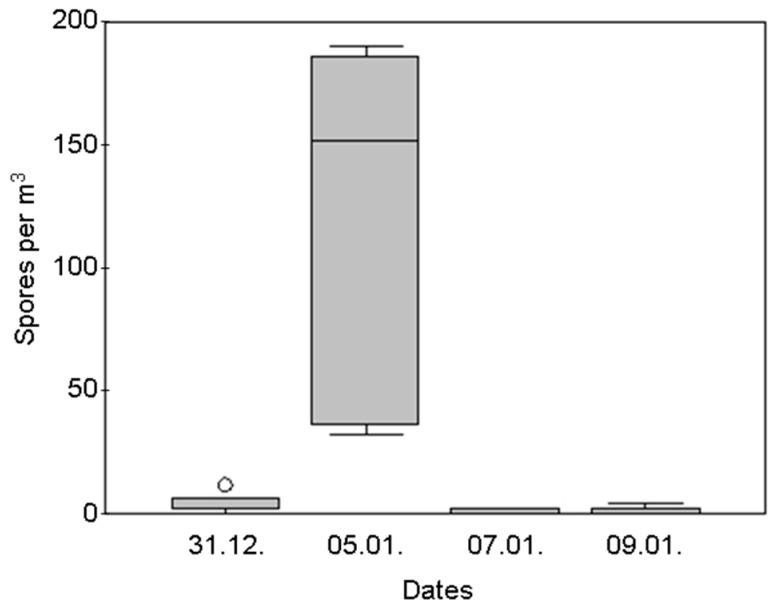
In-house *Aspergillus* spore concentration before, during (5 January) and after removal of the Christmas-trees.

*Aspergillus* spores peaked with a median of 148 spores/m^3^ (IQR 48–177) on 5 January immediately after removing Christmas trees, decoration and cleaning work (Figure 3). The rise in spore counts was transitory and air sampling 48 h later showed a normal count of <3 *Aspergillus* spores per m^3^ at each sampling site. The spore concentrations of the other fungi genera were not affected by Christmas decoration.

## 4. Discussion

We monitored a low and constant level of *Aspergillus* spores in in-house air throughout the year comparable to other studies done in hospitals [8,16]. Contrary to the annual low level, spores of *A. fumigatus* can increase temporary to a significantly high concentration and then decline to normal level within 30 min only [13]. We also have monitored such an increase of *Aspergillus* spores after the removal of Christmas trees. The sedimentation rate of spores of <2 µm is 0.01 to 0.1 cm/s (in calm air, at 0 °C and 1000 hPa) leading to a clearance of one cubic meter air in 3 to 30 h [17]. Due to its hydrophobic surface, *A. fumigatus* spores are dispersed very efficiently by air agitation [18]. It is very likely that peak concentrations of *Aspergillus* spores were often missed during monitoring studies, explaining why it is so difficult to determine cut-off concentrations for increased risk of IA [19]. The annual concentration of spores, the exposure to temporary peak concentration, the time of exposure, and a changed behavior of susceptible patients in summer compared to winter [20] are environmental factors that influence the risk of IA. Among the thermotolerant aspergilli, >95% were identified as *A. fumigatus,* which are described as the main cause for IA. Other thermotolerant *Aspergillus* spp. were not further specified as these play a minor role for IA. Its ability to grow at body temperature, the efficient dispersal of the spores, and its numerous virulence genes [18,21] makes *A. fumigatus* a potent pathogen in immune-suppressed patients.

The distinct seasonality of the remaining fungal spore is mainly due to the dominance of *Cladosporium* spores during summer. Its association with air temperature was concordantly described [8,17], as well as its influence on indoor air spore concentration [4,8,22,23]. The indoor relative humidity paralleled the temperature measured outdoor, reflecting the dependence of the water saturation from air temperature. Interestingly, neither the outdoor measured relative humidity (monthly means were above 80% from September to February and below 80% in the remaining month) nor precipitation showed a correlation with the fungal spore counts. 

Spore concentration can vary dramatically in a building, between different sampling sites, demonstrating that spores aggregate locally. The different sampling sites were close to each other and not separated, enabling fee air-flow between them. Factors, such as air-draft in the corridors, open windows or doors, and activity of people, were not investigated in detail for the present study. The ventilation system of the hospital aerates the HEPA-filtered high-pressure unit at the 4th floor only. The rooms, corridors and the staircase are aerated via the hospital entrance, open windows, and room doors. This makes it difficult to locate sources of spore contamination. Therefore, we have only for two sites an explanation for the diverse spore frequency: (i) the low spore concentration in the recreation area at the 4th floor near the HEPA-filtered high-pressure unit can be explained with a thinning of the spores with filtered air that flows out of this unit. This finding shows the effectiveness of HEPA filter equipment to reduce spores. (ii) The frequently open doors of the main entrance, as well as the windows (essentially during summer time), led to an intermixing of the outside air in the basement. The spore concentration of the outdoor air was described to be higher than the indoor air, explaining the observed high concentration of total, as well as of *Aspergillus* spores, in the basement [4,7,8,22].

For monitoring studies, we urgently suggest a high sampling frequency and to include multiple sampling sites. The high inter-sampling variability (see the different recovery rates in Figure 2) should be considered for airborne pathogen sampling studies. This variability is especially high for the recovery of *Aspergillus* spore: only 57% out of all 624 plates collected during the study showed growth of *Aspergillus* spp.

## 5. Conclusions

We established a reference base-line for spore frequency in a noncomplaint hospital building (*i.e.*, without mould-related problems) using a data set with 624 records of airborne spores collected within one year. Such data are important to recognize increases of spore concentration (e.g., decoration activities during Christmas time). The seasonal fluctuation of spore concentration measured indoors highlighted the impact of environmental conditions on air quality in hospital buildings. The observed inter-sampling variability emphasizes the importance of having multiple sites, as well as an appropriate observation period (e.g., several weeks) for monitoring studies.
